# Prognostic value of high-sensitivity cardiac troponin in non-cardiac surgical patients in intensive care units

**DOI:** 10.1007/s11739-023-03509-z

**Published:** 2024-01-09

**Authors:** Jitain Sivarajah, Michael Toolis, Samantha Seminoff, Jesse Smith, Vikram Bhalla, Eldho Paul, Ravindranath Tiruvoipati

**Affiliations:** 1 Department of Intensive Care Medicine, Frankston Hospital, 2 Hastings Road, Frankston, VIC 3199 Australia; 2 Department of Intensive Care Medicine, Dandenong Hospital, 135 David Street, Dandenong, VIC 3175 Australia; 3 Faculty of Medicine, Nursing and Health Sciences, Monash University, Melbourne, Australia; 4 ANZIC-RC, School of Public Health and Preventive Medicine, Monash University, 553 St Kilda Road, Melbourne, VIC 3004 Australia; 5 Peninsula Clinical School, Monash University, Faculty of Medicine, Nursing and Health Sciences, Frankston, Australia

**Keywords:** Myocardial infarction, Cardiac troponin, Mortality, Surgery, Intensive care

## Abstract

Type II myocardial injury following surgical procedures is associated with adverse outcomes. The prognostic value of high-sensitivity cardiac troponin (hs-cTn) due to type II myocardial injury in surgical patients admitted to intensive care unit (ICU) remains unclear. The aim of this study was to assess prognostic value of hs-cTn in type II acute myocardial injury in non-cardiac surgical patients requiring post-operative ICU admission. Retrospective analysis of patients admitted to two level III ICUs following surgery and had hs-cTn measured on the day of ICU admission. Patients who had type I acute myocardial infarction (AMI) during their admission were excluded from the study. The primary outcome was hospital mortality. Secondary outcomes included ICU mortality, ICU length of stay (LOS) and hospital LOS. A total of 420 patients were included. On univariable analysis, higher hs-cTn was associated with increased hospital mortality (14.6% vs 6.3%, *p* = 0.008), ICU LOS (41.1 h, vs 25 h, *p* = 0.004) and hospital LOS (253 h vs 193 h, *p* = 0.02). On multivariable analysis, hs-cTn was not independently associated with increased risk of hospital mortality. However, in patients who had elective surgery, hs-cTn was associated with increased risk (OR 1.048; 95% CI 1.004–1.094; *p* = 0.031) of hospital mortality with area under the receiver operating characteristic curve of 0.753 (95% CI 0.598–0.908). In elective surgical patients, hs-cTn was associated with increased risk of mortality. Larger multicentre studies are required to confirm this association that may assist in risk stratification of elective surgical patients requiring ICU admission.

## Background

Many patients are admitted post-operatively to intensive care units (ICU) after undergoing both elective and emergency surgery [1]. The goal of such post-operative admissions to ICU as opposed to regular hospital ward locations is to provide an environment which optimises patient outcomes by allowing clinicians to closely monitor, rapidly identify and act upon evidence of patient deterioration [2, 3].

Myocardial injury, defined pathologically as myocardial cell death due to prolonged ischaemia, clinically denotes the presence of acute myocardial injury as detected by abnormal cardiac biomarkers in the setting of evidence of acute myocardial ischaemia [4]. Mortality related to myocardial injury is one of the leading causes of postoperative death within 30 days of non-cardiac surgery [5, 6]. The mortality is mostly due to perioperative acute myocardial infarction (AMI) due to myocardial injury caused by coronary artery diseases including plaque rupture or thrombosis (type I AMI) or due to supply–demand mismatch (type II AMI) due to several post-operative conditions including hypotension, tachycardia, hypoxemia and sepsis [7]. Blood measurements of high sensitive cardiac troponin (hs-cTn) are a well-known predictor of mortality and other adverse patient outcomes in multiple settings, including ICU [8–12]. The predictive value of cardiac troponin in the post-operative phase has been studied in multiple subsets of post-operative surgical patients [13–15]. These studies including patients with type I and type II AMI reported that cardiac troponin or hs-cTn had independent association of increased risk of mortality. High sensitive cardiac troponin assays detect changes with lower levels of troponins more easily as compared to the more traditional troponin assay. While this increased sensitivity plays an important role in patients with type I AMI, it is uncertain at this stage as to how hs-cTn are to be interpreted in the setting of type II AMI during perioperative period.

While the treatment options of type I AMI are well established targeting revascularisation, the management of type II AMI in perioperative setting is evolving. The management is largely supportive care aimed to correct the imbalance between oxygen supply—demand mismatch [16]. It is unknown if hs-cTn predicts short-term mortality in type II AMI post-operative patients as none of the earlier studies specifically investigated this group of patients. Furthermore, earlier studies included patients managed in surgical wards where vital signs are evaluated only every 4–8 h contrasting to continuous monitoring of patients intraoperatively or in intensive care units post-operatively [6, 13, 17].

To the best of our knowledge, the role of hs-cTn in type II myocardial injury and in patients admitted specifically to ICU (where patients are monitored continuously) following elective and emergency surgery has not yet been studied.

Our study aimed to investigate the association of hs-cTn due to type II myocardial injury with hospital mortality in non-cardiac surgical patients admitted to ICU following both elective and non-elective surgery.

## Methods

### Ethical considerations

Ethics approval was obtained from the Human Research and Ethics Committee of Peninsula Health (Reference number QA/69001/PH-2020-234264) and Monash Health (Reference number QA/71890/MonH-2020-241855). Informed consent was waived by ethics committees as data was already collected as part of routine quality assurance processes.

Postoperative patients admitted to ICU over a 4-year period from April 2016 to May 2020 which corresponded to the introduction of hs-cTn measurements at our study sites were screened. Patients were included in the study if they were admitted to ICU following elective or emergency surgery and had a hs-cTn assay on the day of admission to ICU. There were no strictly defined criteria for testing of hs-cTn in ICU, but it was generally performed in patients older than 60 years, had cardiovascular comorbidities or were requiring vasoactive agents. Patients were excluded if they had been diagnosed with an acute myocardial infarction perioperatively or during their current hospital admission. Patients included were stratified into two groups based on their serum hs-cTn: a low troponin group (< 15 ng/L in females and < 33 ng/L in males) and a high troponin group (> 15 ng/L in females and > 33 ng/L in males). These values were based on the normal reference range in our laboratories. The hs-cTn values were assayed with UniCel DxI 800 platform (Beckman Coulter). The upper reference limit was 15 ng/L in females and 33 ng/L in male patients. Data were collected from our ICU databases, hospital pathology databases and individual patient case records.

Data on physiological, laboratory variables and scores derived from scoring systems (American Society of Anaesthesiologists physical status classification system [ASA] and acute physiology age and chronic health evaluation III score [APACHE III]) during the first 24 h were collected, and the most abnormal values during the first 24 h were analysed. The biochemical variables analysed included high-sensitivity troponins, lactate, sodium, potassium, blood glucose, haemoglobin, white cell count, platelet count, bilirubin, albumin, creatinine and urea. Physiological variables included age, sex, heart rate, blood pressure (systolic, diastolic and mean), respiratory rate, temperature, Glascow Coma Scale, FiO2 requirements, PaO_2_, PaCO_2_, pH, HCO_3_^–^, whether or not invasive or non-invasive ventilation was used and whether or not inotropes or vasopressors were used during the first 24 h of ICU admission.

### Patient management in ICU

All patients admitted to ICU had fixed patient to nurse ratio depending on the monitoring and treatments required. Patients were nursed 1:1 if the patients required mechanical ventilation or had haemodynamic instability that required vasoactive medications and 1:2 otherwise. All patients had continuous monitoring of ECG, oxygen saturation, blood pressure and respiratory rate during their ICU stay. All patients had ECG on admission to ICU and at least once daily while in ICU. Over 90% of the patients had invasive haemodynamic monitoring using intra-arterial catheters.

The primary outcome was in-hospital mortality. The secondary outcomes included ICU mortality, ICU and hospital length of stay, development of acute renal failure and in patient cardiac arrest. ARF was defined as a 24 h urine output < 410 ml and serum creatinine ≥ 133 μmol/L and no chronic dialysis.

### Statistical analysis

All analyses were performed with SAS software version 9.4 (SAS Institute, Cary, NC, USA). Baseline and outcome variables were compared between groups (high vs low hs-cTn) using chi-square tests or Fisher’s exact tests, as appropriate, for categorical variables; Student’s t tests for normally distributed continuous variables; and Wilcoxon rank sum tests otherwise, with results presented as frequency (proportion), mean (SD), and median (interquartile range [IQR]), respectively. Univariable and multivariable analyses for hospital mortality were performed using logistic regression modelling with results presented as odds ratios (OR) and 95% confidence intervals (95% CI). Variables with a p < 0.05 on univariable analysis or those deemed to be clinically relevant were considered for inclusion in the multivariable regression model. The variables included in the final model were highest heart rate, APACHE III score and highest hs-cTn on day of admission to ICU. The interaction between hs-cTn and type of surgery (elective vs emergency) was assessed by fitting main effects for hs-cTn, type of surgery and their two-way interactions. The prognostic value of hs-cTn in predicting hospital mortality was assessed by calculating area under the receiver operating characteristic curves (AUROC). The AUROC was interpreted as follows: 0.9–1, high accuracy; 0.7–0.9, moderate accuracy; 0.5–0.7, low accuracy and 0.5 a chance result [18]. The optimal cut-off point for hs-cTn to predict hospital mortality was determined using Youden’s index [19, 20]. Troponin was analysed as a continuous variable in all regression analyses. All calculated p values were two-tailed and *p* < 0.05 indicated statistical significance.

## Results

**Fig. 1:**
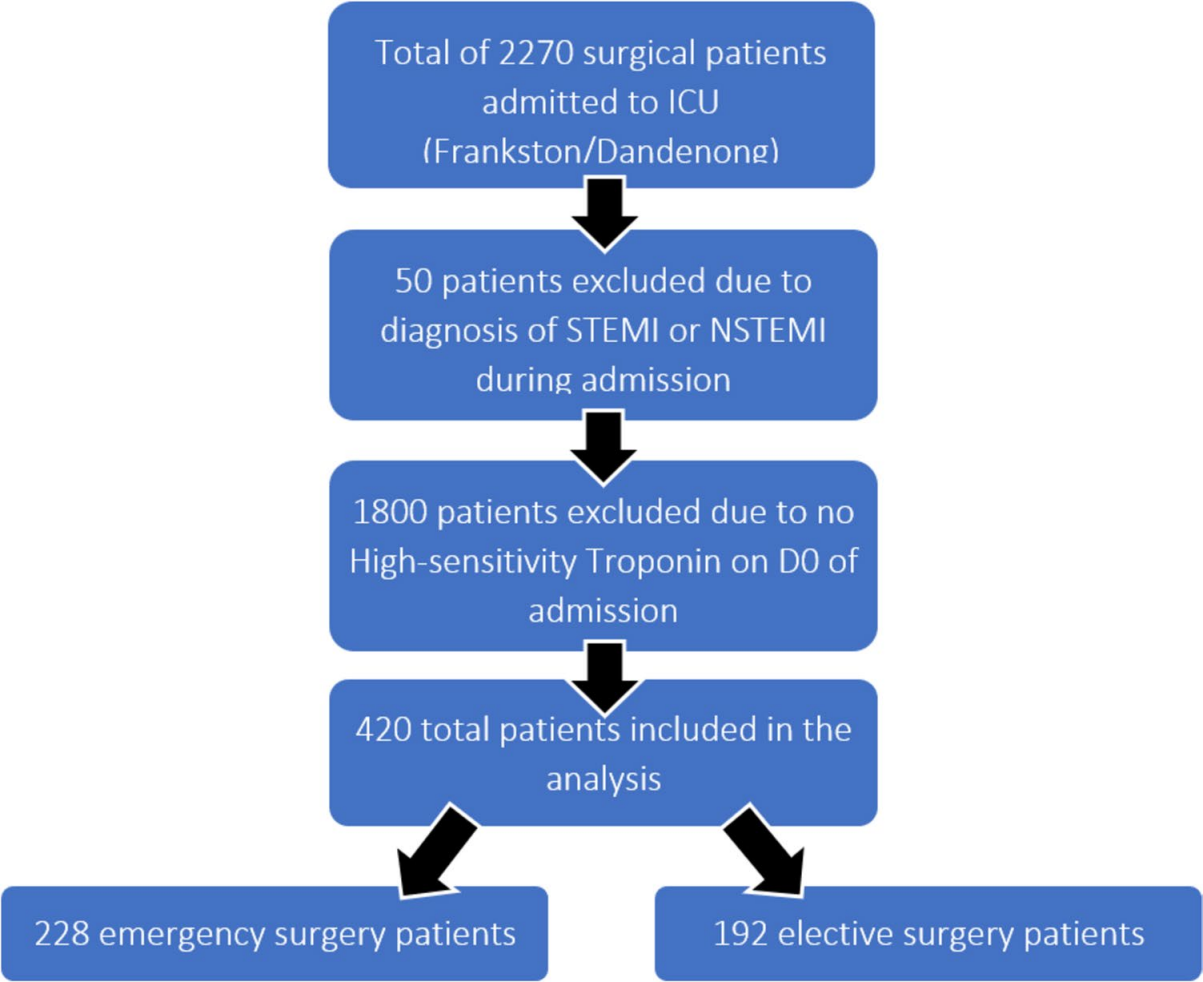
Study Profile

During the study period, a total of 2270 patients were admitted to ICUs post-operatively following either emergency or elective surgical procedures. Of these patients, 420 (18.5%) were included in the analysis. 1850 patients were excluded due to a diagnoses of acute myocardial infarction during their admission (*n* = 50) and troponins were not measured within the first 24 h of ICU admission in 1800 patients. Inclusion and exclusion of patients in the study are presented in Fig. 1.

24% (103/420) of patients showed an elevation in hs-cTn during the first 24 h of their ICU admission. Table 1 presents a comparison of demographics, physiological and biochemical characteristics of patients with elevated and normal hs-cTn. Patients with elevated hs-cTn had a median of 53 ng/L [36–146] as compared to normal 6 ng/L [3.2–12] (p < 0.001).

**Table 1:** Comparison of demographical, physiological and biochemical characteristics at time of admission of patients to Intensive Care

Variable	Normal-Troponin group (*n* = 317)	Elevated-Troponin group (*n* = 103)	*p* value
Age (*n* = 420)	71.2 [60.5–79.1]	76.5 [65.3–83.7]	0.004
Sex, M (*n* = 420)	68.8% (218)	46.6% (48)	< 0.0001
Comorbidities			
Cardiovascular disease (*n* = 420)	2.2% (7)	0.971% (1)	0.43
Respiratory disease (*n* = 420)	1.6% (5)	3.9% (4)	0.16
Liver disease (*n = *420)	2.2% (7)	4.9% (5)	0.16
Renal disease (*n* = 420)	1.3% (4)	2.9% (3)	0.37
Immunosuppressed (*n* = 420)	2.2% (7)	5.8% (6)	0.07
Metastatic malignancy (*n* = 420)	2.5% (8)	2.9% (3)	0.83
Elective surgery (*n* = 419)	61.1% (193)	34% (35)	< 0.0001
Vital signs			
Heart rate (/min) (*n* = 420)	93.5 (19.2)	98.4 (22.4)	0.028
Systolic Blood Pressure (mmHg) (*n* = 420)	150 (20)	148 (24.6)	0.45
Diastolic Blood Pressure (mmHg) (*n* = 420)	70 [63–82]	68 [60–80]	0.038
Mean Blood Pressure (mmHg) (*n* = 420)	97 [88–106]	93 [85-104]	0.045
Respiratory Rate (/min) (*n* = 415)	22.4 (4.29)	23.4 (5.61)	0.07
Temperature (°C) (*n* = 420)	36.6 (0.697)	36.8 (0.785)	0.042
Glascow Coma Scale *(n* = 414)	14.6 (0.969)	14.5 (1.12)	0.5
FiO_2_ Requirements (*n* = 389)	0.28 [0.21–0.3]	0.3[0.25–0.45]	<0.0001
Severity of illness			
ASA (*n* = 391)	3 [3-4]	3 [3-4]	< 0.0001
APACHE III (*n* = 418)	50.2 (16.3)	65 (21.4)	< 0.0001
Inotropes used (D_0_) (*n* = 406)	22.7% (70)	39.8% (39)	0.001
Invasive ventilation (D_0_) (*n* = 289)	24.7% (53)	45.9% (34)	0.001
Non-invasive ventilation (*n* = 284)	5.7% (12)	5.6% (4)	0.97
Investigations			
Lactate (*n* = 397)	1.4 [1–2.5]	2 [1.3–3.5]	< 0.0001
pH (*n* = 389)	7.37 (0.067)	7.34 (0.09)	< 0.0001
paO_2_ (mmHg) (*n* = 389)	77 [69–100]	78 [69–120]	0.37
paCO_2_ (mmHg) (*n* = 389)	40.2 (6.51)	39.9 (8.77)	0.77
HCO_3_^−^ (mmol/L) (*n* = 415)	23.3 (3.36)	22 (4.29)	0.001
Sodium(mmol/L) (*n* = 418)	137 (3)	137 (3.99)	0.14
Potassium (mmol/L) (*n* = 415)	4.26 (0.465)	4.19 (0.639)	0.22
Blood Glucose (mmol/L) (*n* = 411)	9.79 (3.24)	10.3 (4.55)	0.18
Haemoglobin (g/dL) (*n* = 418)	10.6 (2.08)	9.69 (1.86)	< 0.0001
White Cells (× 10^9^/L) (*n* = 417)	13.9 (6.5)	15.8 (9.46)	0.031
Platelets (× 10^9^/L) (*n* = 417)	224 (109)	207 (90)	0.17
Serum Bilirubin (µmol/L) (*n* = 415)	12 [19-17]	13 [9-12]	0.23
Serum Albumin (g/L) (*n* = 417)	28.6 (5.58)	25.4 (5.56)	< 0.0001
Creatinine (µmol/L) (*n* = 418)	82 [66–106]	112 [74–204]	< 0.0001
Urea (mmol/L) (*n* = 418)	7 [5.3–9.5]	10.4 [6.9–17.5]	< 0.0001

Data presented with mean and standard deviation for Heart Rate, Respiratory Rate, Temperature, Systolic BP, GCS, APACHE III, pH, PaCO_2_, HCO_3_^−^, Sodium, Potassium, Blood Glucose, Haemoglobin, White Cells, Platelets and Albumin and with median and interquartile ranges unless specified. Vital signs presented as mean or median of highest recorded

*n*  number of patients where data were available for analysis, *D*_*0*_ day of admission, *ARF* acute renal failure, *LOS* length of stay.

Patients who had emergency surgery had a higher elevation of hs-cTn (13 ng/L [6–39] vs 7 [3.1–16.5]; *p* < 0.001) as compared to elective surgical patients and had a higher ASA score (3[3–4] vs 3[3–3]; *p* < 0.01) and APACHE III scores (Mean 59.4 (SD 20.5) Vs 49.1 (15.9); *p* < 0.001).

Table 2 presents the characteristics of patients who survived to hospital discharge versus those who died during their admission. Patients who died in hospital were older, had higher proportion of emergency surgery, higher ASA and APACHE III score and the need for inotropes and invasive mechanical ventilation.

**Table 2:** Characteristics of patients who died in hospital and survived to hospital discharge

Variable	Survived to hospital discharge (*n* = 385)	Died in hospital (*n* = 35)	*p* value
Age (*n* = 420)	71.3 [60.5–79.4]	79.2 [72.3–83.7]	< 0.0001
Sex, M (*n* = 420)	63.2% (244)	62.9% (22)	0.97
Comorbidities			
Cardiovascular disease (*n* = 420)	1.6% (6)	5.7% (2)	0.08
Respiratory disease (*n* = 420)	2.3% (9)	0% (0)	1
Liver disease (*n* = 420)	2.6% (10)	5.7% (2)	0.29
Renal disease (*n* = 420)	1.3% (5)	5.7% (2)	0.05
Immunosuppressed (*n*=420)	3.1% (12)	2.9% (1)	0.93
Metastatic malignancy (*n* = 420)	2.3% (9)	5.7% (2)	0.23
Elective surgery (*n* = 420)	56.6% (218)	28.6% (10)	0.001
Vital signs			
Heart rate (/min) (*n* = 420)	93.1 (19.3)	111 (21.7)	< 0.0001
Systolic blood pressure (mmHg) (*n* = 420)	150 (20.6)	145 (26.8)	0.19
Diastolic blood pressure (mmHg) (*n* = 420)	70 [63–82]	66 [60–75]	0.09
Mean blood pressure (mmHg) (*n* = 420)	96 [87–106]	92 [82–103]	0.1
Respiratory rate (/min) (*n* = 416)	22.5 (4.41)	24.2 (6.64)	0.037
Temperature (°C) (*n* = 420)	36.6 (0.684)	36.8 (1.05)	0.18
Glascow Coma Scale (*n* = 415)	14.5 (1.02)	14.5 (0.887)	0.79
FiO2 requirements (*n* = 389)	0.28 [0.21–0.39]	0.3 [0.21–0.7]	0.07
Severity of illness			
ASA (*n* = 392)	3 [3-4]	4 [3-4]	< 0.0001
APACHE III (*n* = 419)	51.7 (17.1)	77 (20.6)	< 0.0001
Inotropes used (D0) (*n* = 407)	24.9% (93)	47.1% (16)	0.005
Invasive ventilation (D0) (*n* = 420)	23.8% (92)	47.1% (16)	0.003
Non-invasive ventilation (*n* = 285)	5.7% (15)	4.3% (1)	0.78
Investigations			
Lactate (*n* = 397)	1.5 [1–2.6]	2.1 [1.2–3.1]	0.05
pH (*n* = 389)	7.36 (0.07)	7.33 (0.107)	0.008
paO2 (mmHg) (n = 389)	78 [69–103]	78 [68–141]	0.85
paCO2 (mmHg) (*n* = 389)	40.2 (6.73)	38.6 (10.3)	0.2
HCO3− (mmol/L) (*n* = 416)	23.3 (3.41)	20.1 (4.89)	< 0.0001
Sodium (mmol/L) (*n* = 419)	137 (3.27)	138 (3.14)	0.049
Potassium (mmol/L) (*n* = 416)	4.55 (0.521)	4.63 (0.767)	0.42
Blood glucose (mmol/L) (*n* = 411)	6.94 (2.4)	6.16 (1.57)	0.06
Haemoglobin (g/dL) (*n* = 419)	10.4 (2.03)	9.66 (2.21)	0.043
White Cells (× 109/L) (n = 418)	14.4 (7.43)	14.1 (6.79)	0.82
Platelets (× 109/L) (*n* = 418)	240 (115)	248 (119)	0.72
Serum Bilirubin (µmol/L) (*n* = 416)	12 [9–18]	17.5 [11–25]	0.003
Serum Albumin (g/L) (*n* = 418)	28.3 (5.47)	22.6 (6.1)	< 0.0001
Creatinine (µmol/L) (*n* = 419)	84 [66–115]	131 [78–251]	0.002
Urea (mmol/L) (*n* = 419)	7.4 [5.7–10.2]	10.4 [8–21.3]	< 0.0001

Data presented with mean and standard deviation for Heart Rate, Systolic BP, Respiratory Rate, GCS, APACHE III, PaCO2, HCO3-, Sodium, Potassium, Blood Glucose, Haemoglobin, White Cells, Platelets and Albumin, and with median and interquartile ranges unless specified. Vital signs presented as mean or median of highest recorded

*n* number of patients where data were available for analysis, *D*_*0*_ day of admission, *ARF* acute renal failure, *LOS* length of stay

On univariate analysis, the primary outcome of in-hospital mortality was higher in elevated hs-cTn group (14.6% Vs 6.3%; *p* = 0.008) (Table 3). Patients in elevated hs-cTn group also had a higher incidence of ARF, inpatient cardiac arrest, longer ICU and hospital LOS. There was a strong trend towards increased ICU mortality in the elevated hs-cTn group, but this did not reach statistical significance (5.8% Vs 2.2%; *p* = 0.07) (Table 3).

**Table 3:** Comparisons of outcomes between patients who had normal troponins and elevated troponins

Variable	Normal-Troponin group (*n* = 317)	Elevated-Troponin group (*n* = 103)	*p *value
Primary outcome			
In-hospital mortality (*n* = 420)	6.3% (20)	14.6% (15)	0.008
Secondary outcomes			
ICU mortality (*n* = 420)	2.2% (7)	5.8% (6)	0.07
ICU LOS(hours) (*n* = 420)	25 [19.8–45]	41.1 [21–71.3]	0.004
Hospital LOS (hours) (*n* = 420)	193 [122–358]	253 [137–460]	0.02
Development of ARF (*n* = 398)	1% (3)	7.2% (7)	0.001
Inpatient cardiac arrest (*n* = 419)	0.316% (1)	2.9% (3)	0.048

Data presented as percentage values and actual values respectively, with media and interquartile range for ICU LOS and Hospital LOS

*ICU* intensive care unit, *LOS* length of stay, *ARF* acute renal failure

**Fig. 2:**
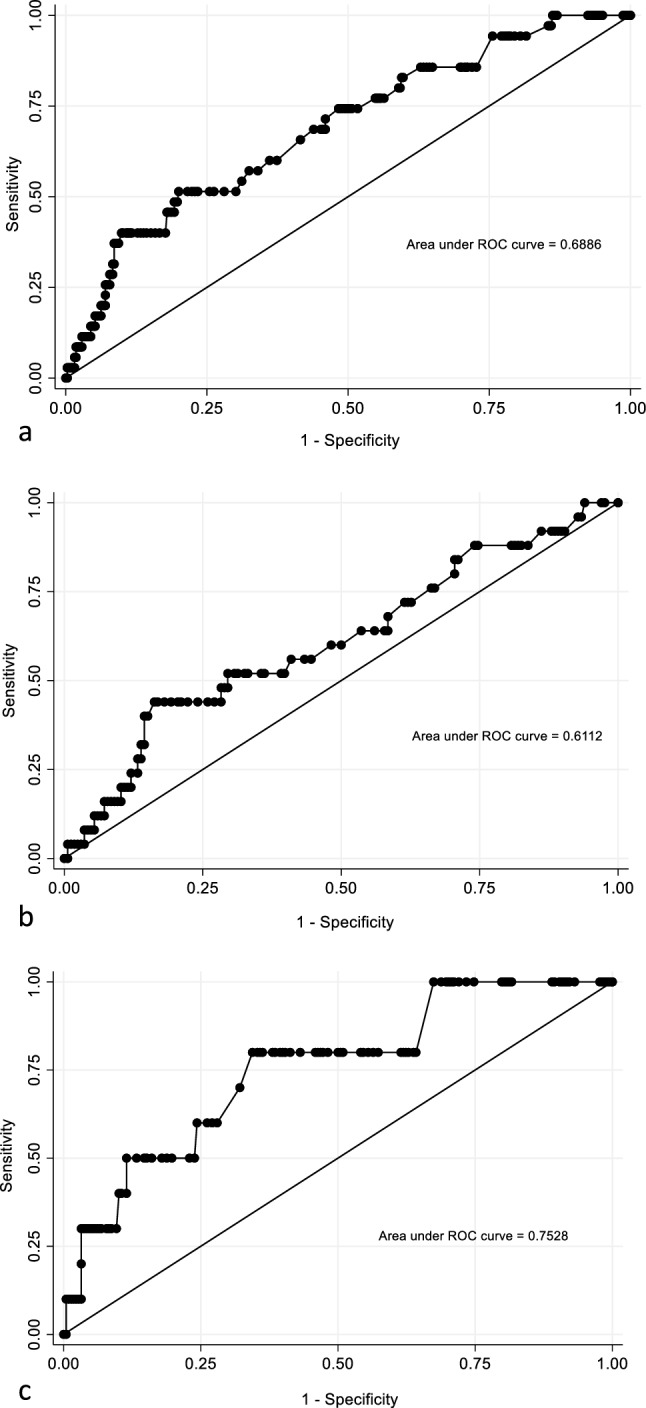
Area under the receiver operating characteristic curves. **a** ROC curve for highest troponin on day 0 (all patients). **b** ROC curve for highest troponin on day 0 (Emergency surgery patients only). **c** ROC curve for highest troponin on day 0 (Elective surgery patients only)

Multivariate logistic regression analysis including all postoperative patients revealed highest heart rate (OR 1.02; 95% CI 1.001–1.04; *p* = 0.036) and APACHE-III score (OR 1.06; 95% CI 1.04–1.08; *p* < 0.0001) were independently associated with hospital mortality. High sensitive cardiac troponin on day of admission to ICU was not found to be an independent predictor of in-hospital mortality (OR 0.998, 95% CI 0.982–1.015; *p* = 0.85). The AUROC (Fig. 2, top panel) for this model was 0.689 (95% CI 0.596 to 0.782). As there was a significant interaction (*p* = 0.049) between hs-cTn and type of surgery (elective vs emergency), analysis was conducted separately for the subgroup of patients who had elective or emergency surgery. This showed that hs-cTn were associated with increased risk of hospital mortality (OR 1.048; 95% CI 1.004–1.094; *p* = 0.031) in elective surgical patients with AUROC of 0.753 (95% CI 0.598–0.908) (Fig. 2, bottom panel), but not in emergency surgical patients (OR 1.003; 95% CI 0.995–1.012; *p* = 0.43; AUROC 0.611, 95% CI 0.485–0.737) (Fig. 2, middle panel). The most favourable combination of sensitivity and specificity for predicting hospital mortality in elective surgical patients was achieved at a hs-cTn threshold of 10. At this cut-off, the sensitivity of hs-cTn was 80% and specificity was 66%.

## Discussion

In this double-centre retrospective study which included over 400 postoperative patients requiring ICU admission at two ICUs after emergency and elective surgery, we aimed to investigate whether high-sensitivity troponin was an independent predictor of in-hospital mortality. After adjusting for confounders with multivariable analysis, high-sensitivity troponin was not found to be independently associated with in-hospital mortality. In the subgroup of patients admitted to ICU after elective surgery, however, elevated high-sensitivity troponins did reveal an association with mortality.

Routine use of high-sensitivity troponins is becoming increasingly common, due to their improved detection of myocardial injury. With their benefit demonstrated in acute coronary syndromes [21] and the role of the routine high sensitive cardiac troponin assay being established as a marker of critical illness in non-cardiac conditions [8, 15], including as an association with mortality, the role of the high-sensitivity troponins in risk stratification of patients undergoing surgery is evolving [7]. In patients after cardiac surgery, high sensitive cardiac troponin were independently associated with increased mortality [22]. Some studies including patients after non-cardiac surgery showed an independent association of high sensitive troponin and increased mortality [13]. However, in studies that specifically included high risk non-cardiac surgical patients, high sensitive troponins were not associated with increased risk of mortality [23].

In our study, overall high sensitive troponins were not independently associated with increased hospital mortality. These results are different to some of the other studies reporting on the association of hs-cTn on mortality in non-cardiac surgical patients [13, 24]. The differences are likely due to the case mix of the patients included as well as the postoperative interventions. The majority of patients develop AMI within 48 h after surgical procedures [5, 16]. Our study included patients who were closely monitored and managed in intensive care units with median duration of ICU stay of about 26 h. This monitoring and management will have prevented or have timely managed hypoxemia or hypotension. Such management is likely to improve outcomes in type II myocardial ischemia.

Nevertheless, our study showed that elevated hs-cTn was associated with mortality in patients undergoing elective surgery but not in patients following emergency surgery. While the cause for an increased risk of mortality in elective surgical patients is not clear from our study, but it highlights the need for closer monitoring of elective surgical patients with elevated hs-cTn. It appears that even smaller elevation of hs-cTn in elective surgical patients is associated with higher risk of hospital mortality.

Our study has multiple strengths worth highlighting. First, ours is the first study to specifically evaluate the prognostic value of post-operative hs-cTn measurements in non-cardiac surgical patients admitted to the ICU post-operatively. Our study specifically investigated patients with type II AMI that is patho-physiologically different from other types of AMI caused by coronary artery disease. Furthermore, we had older patients (median age 72.2 year IQR 61.3–79.9 years) in our study as compared to other studies that had younger patients [13]. Older patients are likely to have more cardiac comorbidities are likely have to have higher risk of cardiac death post-operatively. Prior studies have addressed post-operative patient populations admitted to either intensive-care or ward-based settings [13] and anaesthetic recovery areas [24] but not specifically to the ICU alone. Our databases allowed us to have clear and robust data collection, and the exclusion of any myocardial infarctions specifically investigates the role of hs-cTn in non-ischaemic (type II) myocardial injury during the perioperative period. The stratification of hs-cTn levels based on the sex of patients provides a more reliable assessment of myocardial injury independent of the sex of the patients included. Another strength of our study is that it was conducted over two different hospitals which are both part of two independent health networks and have slightly different surgical case mixes. We expect this study will be generalisable to similar centres.

Our study also has weaknesses. Firstly, our study was retrospective by design and hence selection bias could not be excluded. Our surgical case mix did also not include trauma patients, or patients undergoing cardiothoracic or neurosurgical procedures who potentially have higher illness severities. We also did not have tested for hs-cTn preoperatively to identify patients with chronic myocardial injury. Moreover, despite a large number of patients screened, only a fraction of eligible patients actually had at least one high-sensitive troponin measured in the first 24 h of their ICU admission (564 in total, including patients excluded as per methodology; 24.8%).

Furthermore, despite our exclusion of patients with a formal diagnosis of T1MI during their admission, it is likely that some patients that were included in this study may have had acute myocardial injury or chronic myocardial injury as opposed to the supply–demand mismatch seen in T2MI.

Important future directions exist for our study. In particular, a larger multicentre prospective study looking at the prognostic value of post-operative hs-cTn in patients admitted to ICUs would yield more conclusive results especially on the role of hs-cTn in elective surgical patients.

## Conclusion

Elevated hs-cTn levels are associated with increased in-hospital mortality in patients admitted to ICU after elective surgery. Larger multicentre studies are required to confirm an association that may assist in risk stratification of elective surgical patients and to further evaluate hs-cTn role in emergency surgical patients requiring post-operative admission to ICU.

## Data Availability

All data generated or analysed during this study are included in this published article.

## Abbreviations

AMI: Acute myocardial infarction
APACHE III: Acute physiology and chronic health evaluation III
ARF: Acute renal failure
ASA: American society of anaesthesiologists (classification system)
CI: Confidence interval
ECG: Electrocardiogram
FiO_2_: Fraction of inspired oxygen
hs-cTn: High-sensitivity cardiac troponin
ICU: Intensive care unit
IQR: Interquartile range
LOS: Length of stay
OR: Odds ratio
SAS: Statistical analysis system
SD: Standard deviation
